# Effect of Xiaoqinglong decoction on gut–lung axis microbiota in allergic rhinitis mice

**DOI:** 10.3389/fmicb.2026.1917709

**Published:** 2026-08-19

**Authors:** Jie Zhang, Si Zhou, Liang-Jing Liu, Xiao-Ling Mao, Luo-Qin Zhang, Hao-Lan Liu, Gan-Long Wu

**Affiliations:** 1 School of Medicine, Jishou University, Jishou, China; 2 People’s Hospital of Jishou City, Jishou, China

**Keywords:** allergic rhinitis, gut microbiota, gut–lung axis, nasal microbiota, Xiaoqinglong decoction

## Abstract

**Objective:**

This study aimed to characterize the effects of Xiaoqinglong decoction (XQLD) on the microbiota of the gut–lung axis at three key sites—the nasal cavity, lungs, and gut—in Allergic rhinitis (AR) mice, with particular attention to site-specific response patterns and their potential links to AR disease resolution.

**Methods:**

An ovalbumin-induced AR mouse model was established, with mice assigned to control, AR, loratadine, and XQLD groups. Intervention outcomes were assessed via behavioral observation, ELISA, histopathology, and 16S rRNA gene sequencing, followed by correlation analysis.

**Results:**

Compared with the AR group, XQLD significantly reduced nasal symptoms, serum OVA-sIgE and IL-4 levels, and nasal mucosal injury. AR induction markedly altered the nasal and gut microbiota, whereas the lung microbiota remained relatively stable. XQLD significantly increased nasal *Oxalobacteraceae* and significantly decreased gut *Rikenellaceae* and *Odoribacter*. Gut *Lactobacillaceae*, *Lactobacillus*, and *Allobaculum* showed upward trends, whereas *Helicobacter* showed a slight downward trend. Several nasal and gut taxa were significantly associated with nasal symptoms and Th2-related inflammatory indicators.

**Conclusion:**

XQLD differentially modulates the gut–lung axis microbiota in AR mice, with the most marked changes observed in the gut, which may correlate with its therapeutic effects and merit further mechanistic exploration.

## 1 Introduction

Allergic rhinitis (AR) is an IgE-mediated Th2-type inflammatory disease of the nose. In recent years, the complex role of the gut microbiota and its metabolites in regulating AR-related immune responses has been gradually revealed. Researchers have defined this interrelationship between the respiratory tract and the digestive tract as the gut-lung axis, which effectively connects the immune systems of the respiratory tract and the gut through circulating immune cells, the shared mucosal immune system, and microbial metabolites (Yang et al., 2025).

During the pathological process of AR, patients exhibit characteristic alterations in the gut microbiota, such as decreased abundances of butyrate-producing genera and *Faecalibacterium*, as well as an imbalanced ratio of Bacteroidetes to Firmicutes. These alterations are closely associated with elevated serum IgE levels and nasal eosinophilic infiltration (Sun et al., 2025). Studies have also confirmed that gut microbiota dysbiosis can directly affect host immune homeostasis. Significant changes in microbial composition can exacerbate the airway inflammatory response to allergens, accompanied by elevated Th2-type cytokines and specific IgE. This process is significantly associated with a reduction in short-chain fatty acid-producing genera and decreased fecal short-chain fatty acid levels (Chen et al., 2022).

Short-chain fatty acids (SCFAs), as core metabolites of microbiota, represent a key component of the gut-lung axis-mediated immune regulation in the respiratory tract. Butyrate, one of the most important SCFAs, on the one hand, promotes regulatory T cell differentiation and inhibits Th2-type inflammatory responses by activating the GPR43/GPR109A signaling pathway; on the other hand, as a histone deacetylase inhibitor, it exerts anti-inflammatory effects through epigenetic regulation. In AR patients, decreased circulating butyrate levels may impair the physiological capacity to suppress allergic inflammation (Yang et al., 2025). Meanwhile, the microbiota participates in maintaining immune homeostasis by regulating the tryptophan-kynurenine-aryl hydrocarbon receptor pathway, and gut microbiota dysbiosis in pediatric AR patients is associated with abnormal activation of this pathway (Wang et al., 2014). Furthermore, there is a bidirectional interaction between the gut microbiota and the nasal microbiota, and together they participate in the pathological process of AR (Dong et al., 2026). Based on the above mechanisms, intervention strategies targeting the gut microbiota have become an important direction in AR treatment research (Hou et al., 2024; Wang C. et al., 2025; Wang L. et al., 2025; Han et al., 2026).

As a traditional Chinese herbal formula commonly used for the treatment of AR, Xiaoqinglong decoction (XQLD) has previously been shown by our research group to alleviate nasal allergic symptoms and inflammatory responses and to partially restore AR-associated alterations in the gut microbiota (Liu et al., 2024; Wang et al., 2024). XQLD contains multiple bioactive compounds, including ephedrine, paeoniflorin, and cinnamaldehyde. Following intestinal delivery, these compounds may undergo microbiota-mediated biotransformation and influence the intestinal microenvironment and microbial metabolic activity, thereby modifying the composition of the gut microbiota (Che et al., 2022; Wang et al., 2014). Systematic reviews have also suggested that traditional Chinese medicines may alleviate chronic airway diseases by increasing short-chain fatty acid production, protecting the intestinal barrier, and regulating inflammatory immune responses (Wei et al., 2025; Zheng et al., 2025). These studies provide a theoretical basis for exploring the treatment of AR by Chinese herbal medicine through the gut-lung axis.

Although previous research has confirmed that XQLD has a certain regulatory effect on the gut microbiota in AR mice, does XQLD also affect the nasal and lung microbiota within the gut-lung axis? Are there differences in its effects on the nasal, lung, and gut microbiota? And is this effect on the gut-lung axis microbiota correlated with its efficacy in alleviating AR symptoms? These issues remain unclear. Therefore, this study simultaneously detected the changes in the nasal, lung, and gut microbiota of AR mice after XQLD intervention and systematically analyzed the correlations between these microbiota changes and AR pathological symptoms, inflammatory cytokine levels, and the degree of mucosal damage repair. The research results preliminarily reveal the overall regulatory characteristics of XQLD on the gut-lung axis microbial community, providing more direct experimental evidence for its clinical application in the treatment of AR.

## 2 Materials and methods

### 2.1 Mouse preparation

Female C57BL/6 mice, aged 6–8 weeks and weighing 18–22 g (Changsha Tianqin Biotechnology Co., Ltd., license number: SCXK (Xiang) 2019-0014). The mice were housed in the Experimental Animal Center of Jishou University School of Medicine. The experimental protocol was approved by the Ethics Committee of Jishou University (approval number: JSDX-2022-0006), and all procedures were strictly conducted in accordance with animal ethical guidelines.

Mice were randomly divided into control group, AR model group, loratadine group, and XQLD group, with 8 mice in each group. An AR mouse model was established by intraperitoneal injection of OVA combined with aluminum hydroxide, followed by intranasal challenge with OVA. Each group was administered by gavage according to the experimental protocol: the XQLD group received XQLD solution, the loratadine group received loratadine solution, and the control group and AR model group received an equal volume of normal saline (Liu et al., 2023, 2024). For detailed procedures, see Supplementary material 1-1.

### 2.2 XQLD preparation

The herbal composition of XQLD is detailed in Supplementary material 1-2, and the botanical names were verified using the Plants of the World Online (POWO) database.^1^ The XQLD solution was extracted using the water reflux method, and the stability of its preparation process as well as the batch-to-batch consistency of its main chemical components were verified by liquid chromatography-mass spectrometry (LC–MS) analysis in previous studies (Liu et al., 2023). In this study, the XQLD group was administered by gavage with a solution at the clinical dose (1×), with a concentration of 2.1 g crude drug/mL, administered by gavage at 100 μL per day, which was also verified as the optimal concentration in previous studies (Liu et al., 2023, 2024).

### 2.3 Evaluation of therapeutic efficacy

To evaluate the therapeutic effect of XQLD in AR mice, the number of sneezes and nose-rubbing episodes in each group was observed and recorded immediately after the final challenge. To avoid operational interference, recording was performed for 10 min starting from the 2nd minute after intranasal instillation, in order to quantify nasal allergic symptoms. The levels of serum OVA-sIgE (Shanghai Jianglai Biotechnology Co., Ltd., Cat. No. JL55065) and IL-4 (Wuhan Elabscience Biotechnology Co., Ltd., Cat. No. E-MSEL-M0008) in mice were measured by ELISA, and all procedures were performed according to the manufacturer’s instructions. Nasal mucosal tissues of mice were collected, fixed in 4% paraformaldehyde, embedded in paraffin, sectioned, and stained with hematoxylin and eosin (H&E). Histopathological changes were observed under a light microscope.

### 2.4 Analysis of nasal, lung, and gut microbiota

Under aseptic conditions, the complete nasal and sinus tissue samples, whole lung tissue samples, and fecal samples of mice in each group were collected. Total DNA was extracted from all collected samples, followed by 16S rRNA gene amplification and sequencing, all performed using the same procedures. Genomic DNA was extracted from samples using a DNA extraction kit. After verifying DNA integrity by gel electrophoresis, the target region (V3–V4 region) of the bacterial 16S rRNA gene was amplified by PCR using a high-fidelity DNA polymerase. After the amplified products were recovered by gel excision and purified, they were precisely quantified by real-time quantitative PCR to ensure that the library quality met the sequencing requirements. High-throughput sequencing was entrusted to Shanghai Personal Biotechnology Co., Ltd. and performed on the Illumina MiSeq platform with 2 × 300 bp paired-end sequencing. The obtained raw sequencing data were subjected to quality control, denoising, assembly, and chimera removal before subsequent analyses of microbial community diversity and structure. Given the low-biomass nature of the nasal and lung samples, the frequency method implemented in the decontam R package was applied using total DNA concentration as the covariate to identify candidate contaminant ASVs (*p*_freq_ < 0.10). Sequence retention rates and major community metrics were compared before and after filtering. Detailed results are provided in Supplementary material 1-3A, 1-3B.

### 2.5 Statistical analysis

Statistical analysis and graph generation in this study were performed using GraphPad Prism 10.1.2, SPSS 23.0, and Adobe Illustrator 2024. Continuous data conforming to a normal distribution were expressed as the mean ± standard deviation ( $\bar{x}\pm s$ ) and visualized using bar charts; non-normally distributed data were expressed as the median and interquartile range [*M* (*P25*, *P75*)] and displayed using box plots. For intergroup comparisons, if the data met the assumptions of normality and homogeneity of variance, one-way analysis of variance (ANOVA) was used, followed by Dunnett’s test for pairwise comparisons; if the conditions for parametric tests were not met, the nonparametric Kruskal–Wallis test was used, supplemented by Dunn’s test for multiple comparisons. The significance level was set at *p* < 0.05. For comparisons of the top 10 dominant taxa at the family and genus levels between the AR and XQLD groups, two-sided Mann–Whitney *U* tests with corrections for ties and continuity were performed. The resulting *p* values were adjusted for multiple comparisons using the Benjamini–Hochberg method within each anatomical site and taxonomic level, with *q* < 0.05 considered statistically significant after false discovery rate correction. Correlations between microbial taxa and immunological indicators were analyzed using Genescloud Tools.^2^

## 3 Results

### 3.1 Modeling and intervention efficacy

**Figure 1:**
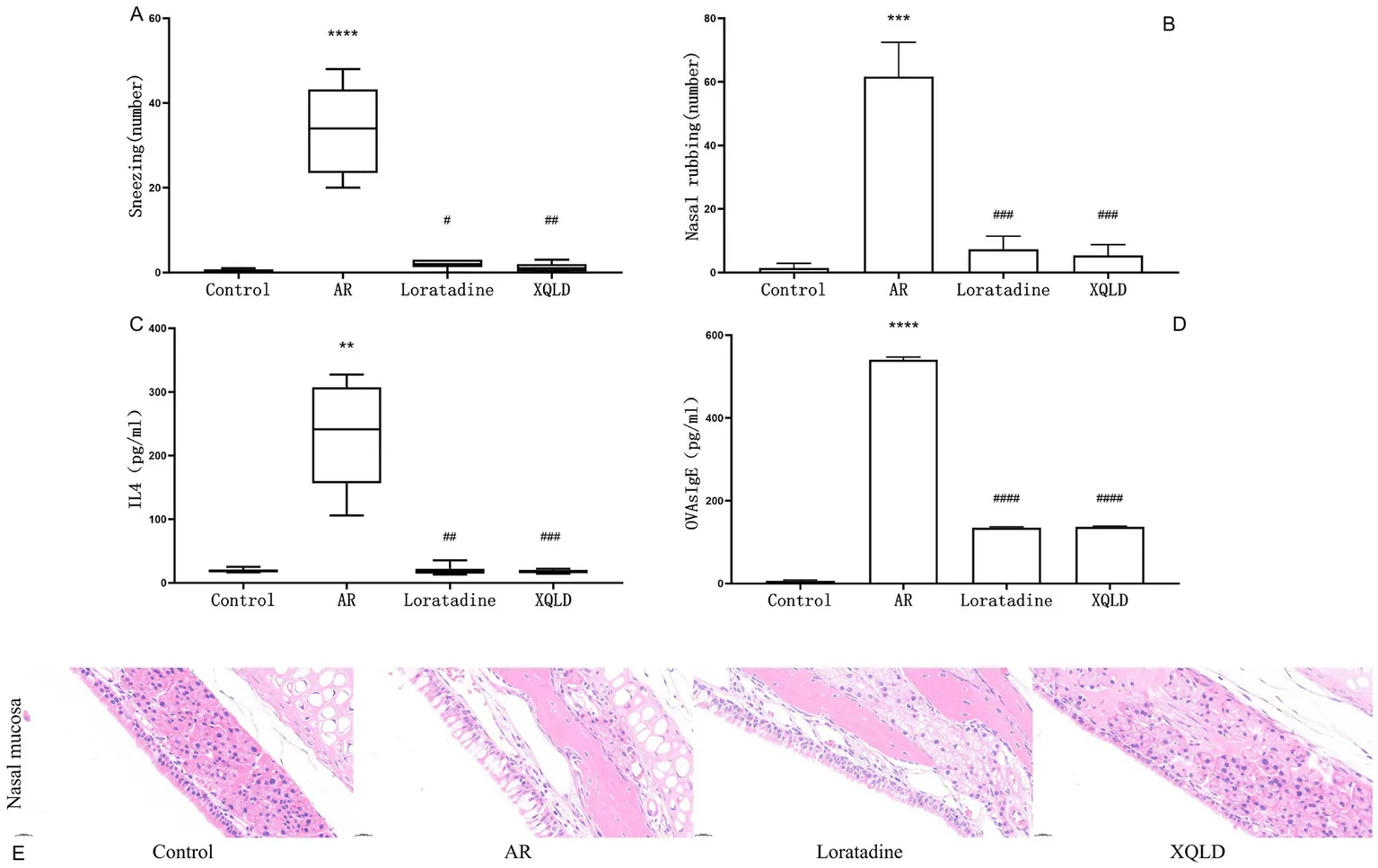
Modeling and drug intervention efficacy in mice (*n* = 8). **(A)** Number of sneezes; **(B)** Number of nose-rubbing episodes; **(C)** Serum IL-4 concentration; **(D)** Serum OVA-sIgE level; **(E)** H&E staining of nasal mucosa. The significance analysis of the AR group is obtained by comparing it with the control group to obtain the *p*-value (^*^p < 0.05 and ^****^p < 0.0001). The significance analysis of the Loratadine and XQLD groups is obtained by comparing them with the AR group to obtain the *p*-value (^#^*p* < 0.05 and ^####^*p* < 0.0001). Control refers to the group where no drug treatment is administered after the modeling process.

In this study, nasal symptoms, inflammatory cytokine levels, and nasal mucosal structural changes were observed (Figure 1). Behavioral observation after the final challenge showed that the numbers of sneezes and nose-rubbing episodes in the AR model group were significantly increased compared with the control group (*p* < 0.05), whereas both the XQLD group and the loratadine group showed significantly decreased behavioral indices compared with the AR model group (*p* < 0.05). ELISA results showed that serum levels of OVA-sIgE and IL-4 in the AR model group were significantly higher than those in the control group (*p* < 0.05), whereas the levels of the above indicators in each treatment group were significantly lower than those in the AR model group (*p* < 0.05). H&E staining of nasal mucosa showed that in the control group, the nasal mucosal epithelium had an intact structure with neatly arranged cilia; in the AR model group, cilia shedding, goblet cell hyperplasia, tissue edema, and inflammatory cell infiltration were observed; in the XQLD group and the loratadine group, the above pathological changes were significantly alleviated, and the nasal mucosa tended to be intact.

### 3.2 Microbiota

#### 3.2.1 Sequencing depth and operational taxonomic units (OTUs)

**Figure 2:**
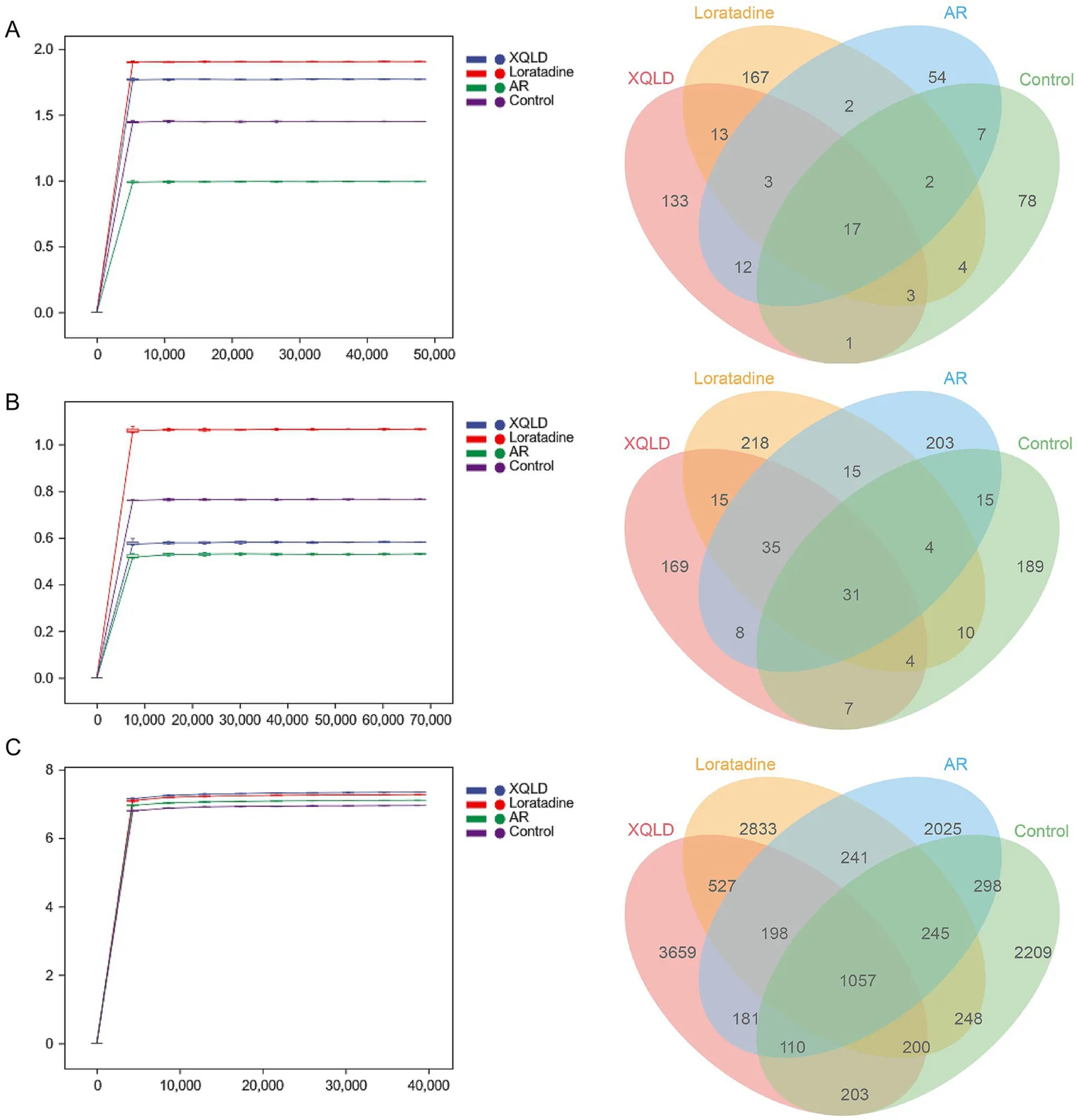
Sequencing depth and OTU distribution of the mouse microbiota. **(A)** Nasal microbiota; **(B)** Lung microbiota; **(C)** Gut microbiota. Rarefaction curves (left) reflect sequencing depth, and Venn diagrams (right) show OTU distribution among the four groups. Control, Control group; AR, AR model group; Loratadine, Loratadine group; XQLD: Xiaoqinglong Decoction group.

To investigate the effects of XQLD on the microbial community structure along the gut–lung axis in AR mice, 16S rRNA sequencing was used to characterize the nasal, lung, and gut microbiota. The rarefaction curves indicated sufficient sequencing depth (Figure 2, left). A total of 489, 742, and 14,234 OTUs were detected in the nasal, lung, and gut microbiota, respectively (Figure 2, right). OTUs shared among the four groups accounted for 3.5, 4.2, and 7.4% of the total OTUs at the respective sites, with all proportions being no greater than 7.4%. Correspondingly, more than 92% of the OTUs at each site were not shared by all four groups, suggesting a high degree of specificity in the microbial composition across anatomical sites and experimental groups. In addition, the numbers of OTUs detected in the nasal and lung microbiota were markedly lower than that in the gut microbiota. Among the treatment groups, the XQLD group contained more unique gut OTUs than the loratadine group, suggesting that the two interventions may exert different effects on gut microbial composition. At a threshold of *p*_freq_ < 0.10, 2 and 4 candidate contaminant ASVs were identified in the nasal and lung samples, respectively. After filtering, more than 99.99% of the sequences were retained in all samples, and the major community metrics and taxonomic profiles at the family and genus levels remained essentially unchanged. Detailed results are provided in Supplementary material 1-3A, 1-3B.

#### 3.2.2 Alterations in the composition of nasal, lung, and gut microbiota

**Figure 3:**
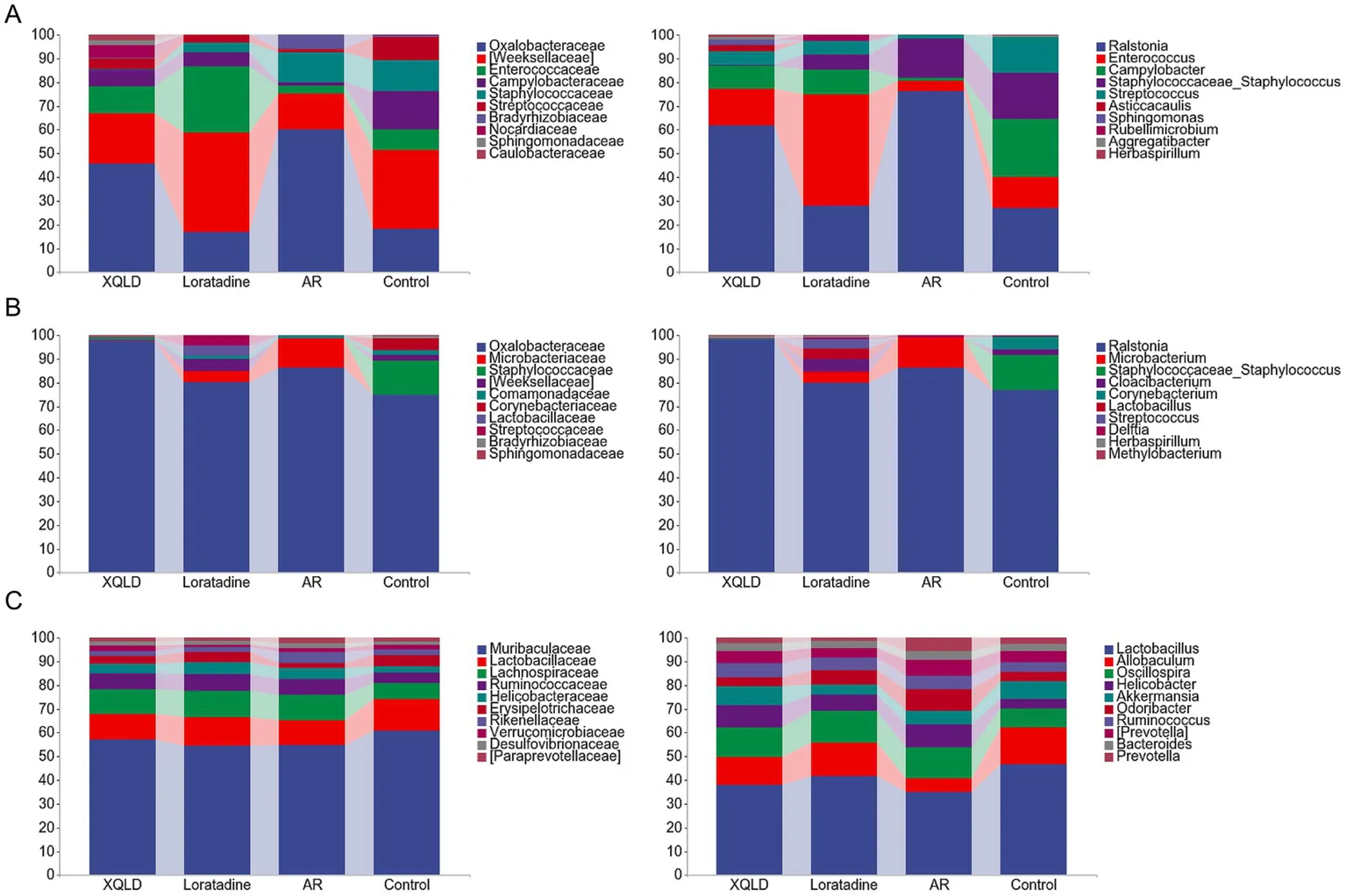
Top 10 taxa ranked by relative abundance of the mouse microbiota at the family level (left) and the genus level (right). **(A)** Nasal microbiota; **(B)** Lung microbiota; **(C)** Gut microbiota. Control, Control group; AR, AR model group; Loratadine, Loratadine group; XQLD, Xiaoqinglong Decoction group.

To characterize the effects of XQLD on the microbiota at different sites along the gut–lung axis in AR mice, we first identified the top 10 most abundant taxa at the family and genus levels in the nasal cavity, lungs, and gut (Figure 3), and then compared their relative abundances between the AR and XQLD groups. AR induction resulted in marked alterations in the nasal and gut microbiota, whereas the lung microbiota remained relatively stable. After FDR correction, three taxa differed significantly between the AR and XQLD groups (*q* < 0.05): nasal *Oxalobacteraceae*, gut *Rikenellaceae*, and gut *Odoribacter* (Table 1).

**Table 1:** Microbial taxa showing significant differences between the AR and XQLD groups after FDR correction.

Site	Taxonomic level	Bacterial taxa	Relative abundance in AR group (%)	Relative abundance in XQLD group (%)	*p* value	*q* value
Nasal	Family	*Oxalobacteraceae*	34.97	39.95	0.0009	0.0094
Gut	Family	*Rikenellaceae*	3.8476	1.6250	0.001359	0.013594
Gut	Genus	*Odoribacter*	2.2131	0.8782	0.001948	0.019475

At the family level (Figure 3, left), the mean relative abundance of nasal *Oxalobacteraceae* was significantly higher in the XQLD group than in the AR group (39.95% vs. 34.97%; *p* = 0.0009, *q* = 0.0094). In the gut microbiota, the mean relative abundance of *Rikenellaceae* was significantly lower in the XQLD group than in the AR group (1.62% vs. 3.85%; *p* = 0.0014, *q* = 0.0136). Gut *Lactobacillaceae* increased from 8.50% in the AR group to 11.61% in the XQLD group, but the difference did not remain statistically significant after FDR correction (*q* = 0.0676). None of the top 10 dominant lung families differed significantly between the two groups (all *q* > 0.05).

At the genus level (Figure 3, right), none of the top 10 dominant nasal or lung genera differed significantly between the AR and XQLD groups after FDR correction (all *q* > 0.05). In the gut microbiota, *Lactobacillus* and *Allobaculum* showed upward trends, whereas *Helicobacter* showed a slight downward trend; however, none of these differences were statistically significant. The mean relative abundance of *Odoribacter* was significantly lower in the XQLD group than in the AR group (0.88% vs. 2.21%; *p* = 0.0019, *q* = 0.0195). Overall, XQLD treatment was associated with taxon-specific changes in the nasal and gut microbiota, whereas the lung microbiota remained relatively stable. Detailed relative abundances and statistical comparisons of the selected taxa are provided in Supplementary material 1-4.

#### 3.2.3 Analysis of nasal, lung, and gut microbiota structural variations

**Figure 4:**
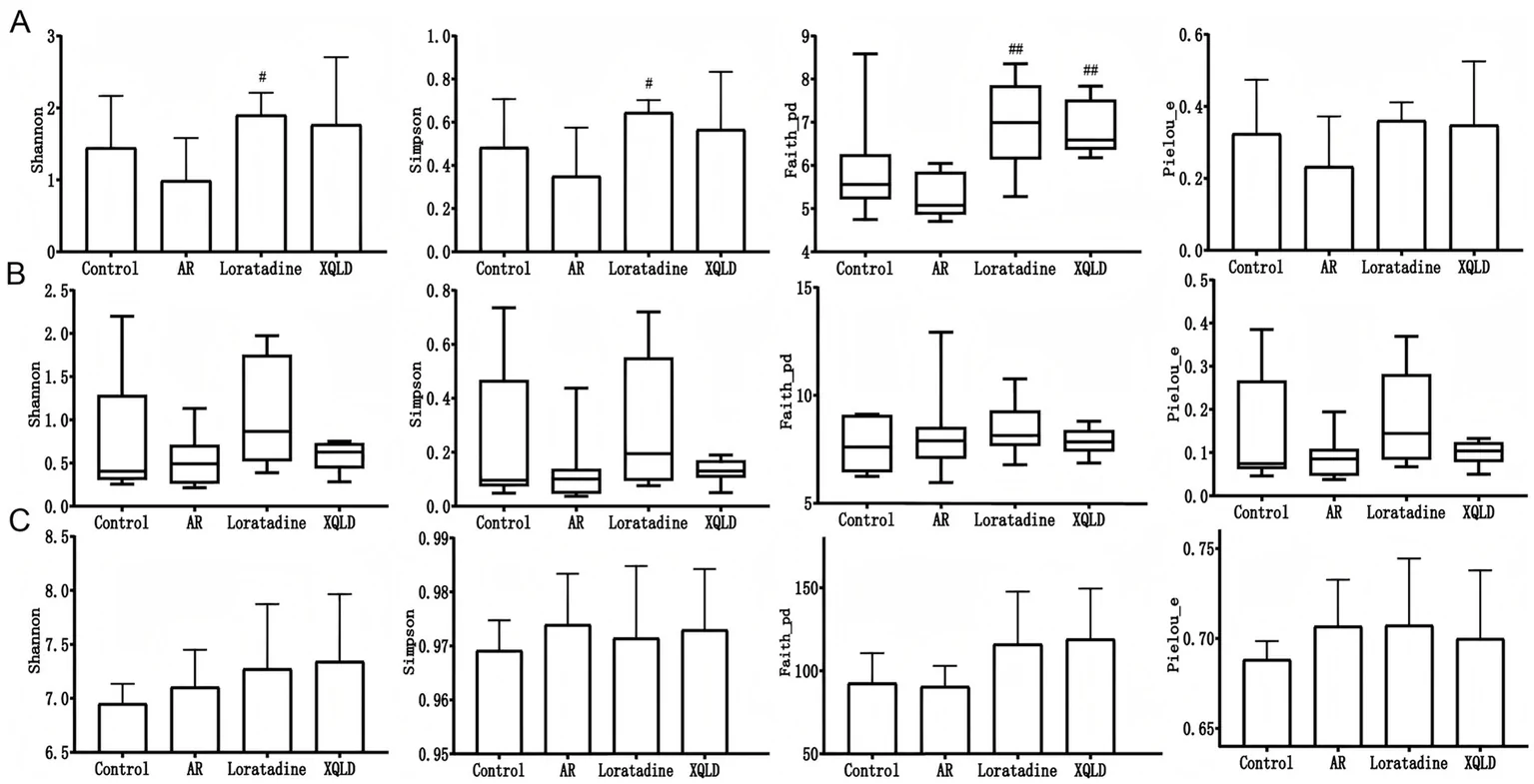
Results of mouse alpha-diversity indices analysis. From left to right are the analysis results of each group for the Shannon diversity index, Simpson diversity index, Faith’s phylogenetic diversity index, and Pielou’s evenness index: **(A)** Nasal microbiota; **(B)** Lung microbiota; **(C)** Gut microbiota. The significance analysis of the AR group is obtained by comparing it with the Control group to obtain the *p*-value (^*^p < 0.05 and ^****^p < 0.0001). The significance analysis of the Loratadine and XQLD groups is obtained by comparing them with the AR group to obtain the *p*-value (^#^*p* < 0.05 and ^####^*p* < 0.0001). Control refers to the group where no drug treatment is administered after the modeling process.

To further explore the differences in the responses of microbiota from different sites of the gut-lung axis to AR modeling and drug intervention, this study comprehensively evaluated the alpha-diversity indices of nasal, lung, and gut microbiota in each group from four dimensions: Shannon diversity index, Simpson diversity index, Faith’s phylogenetic diversity index, and Pielou’s evenness index (Figure 4). Analysis showed that the response patterns of alpha-diversity indices of microbiota in different sites to AR modeling and drug intervention were markedly different.

Nasal microbiota analysis (Figure 4A) showed that the Shannon, Simpson, and Faith’s phylogenetic diversity indices in the AR group exhibited a decreasing trend. Following loratadine or XQLD treatment, all diversity indices showed an upward trend, with Faith’s phylogenetic diversity index being significantly increased compared with the AR group (*p* < 0.05). These findings suggest that both treatments may alleviate the decline in phylogenetic diversity of the nasal microbiota associated with AR. Regarding the lung microbiota (Figure 4B), there were no statistically significant differences in the four alpha-diversity indices among the groups (*p* > 0.05), indicating that the AR model group and drug intervention had little effect on the diversity of the lung microbiota, a result consistent with the species composition analysis. Regarding the gut microbiota (Figure 4C), there were no significant differences in alpha-diversity indices between the AR model group and the control group (*p* > 0.05); only the Faith index approached the critical value between the AR model group and the XQLD group (*p* = 0.0966). Overall, the alpha-diversity indices of the gut microbiota remained relatively stable among all groups.

**Figure 5:**
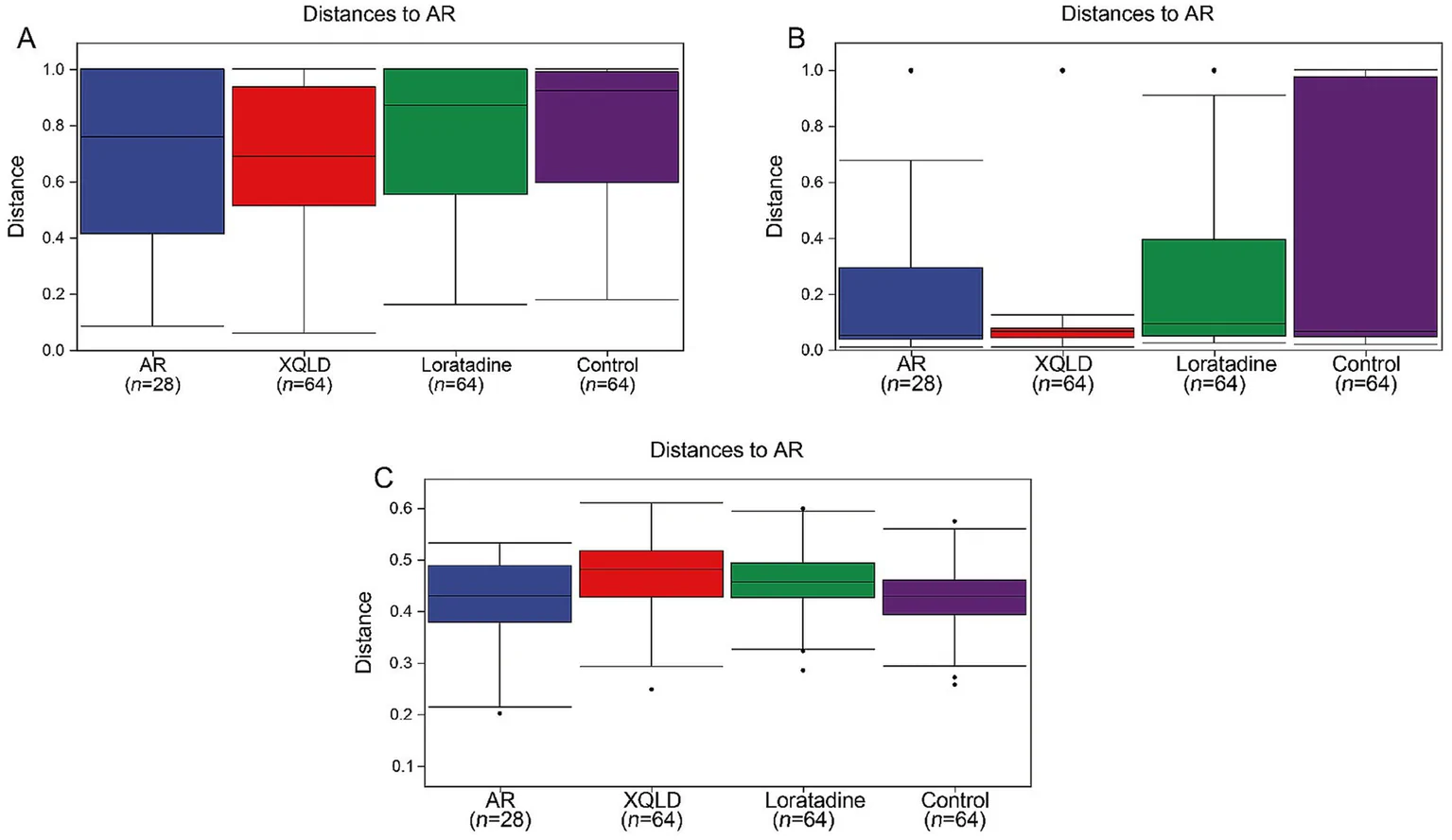
Beta diversity distance analysis of mouse microbiota. **(A)** Nasal microbiota; **(B)** Lung microbiota; **(C)** Gut microbiota. Box plots show the distribution of beta diversity distances between each group and the AR model group; larger distances indicate greater differences in community structure. The sample size for each experimental group is 8. The *n* values shown below each group denote the number of distance values used for statistical analysis: AR group *n* = 28 (within-group pairwise comparisons among 8 samples [*C*(8, 2) = 28]); XQLD, Loratadine, and Control groups *n* = 64 (between-group pairwise distances comparing 8 samples from each group with 8 samples from the AR group [8 × 8 = 64]). Box plot elements: Box bounds = Q3 and Q1; midline = median; whiskers = min to max within 1.5 × IQR; dots beyond whiskers = outliers. Control, control group; AR, allergic rhinitis model group; Loratadine, loratadine-treated group; XQLD, Xiaoqinglong Decoction-treated group.

To further evaluate the overall differences in microbiota structure among different groups, beta diversity analysis was performed in this study (Figure 5). The results showed that the response patterns of nasal and gut microbiota to the AR model group and drug intervention differed significantly among groups, whereas no significant difference was observed in the lung microbiota.

For the nasal microbiota (Figure 5A), there was a significant overall difference among groups (*r* = 0.113, *p* < 0.05). For pairwise comparisons, the community structure difference between the loratadine group and the AR model group was relatively significant (*r* = 0.297, *p* < 0.05), whereas no significant difference was observed between the XQLD group and the AR model group. Regarding the lung microbiota (Figure 5B), there was no significant overall difference among groups (*r* = 0.041, *p* > 0.05). For the gut microbiota (Figure 5C), there was a significant overall difference among groups (*r* = 0.134, *p* < 0.05). Pairwise comparisons revealed significant differences between the AR model group and the control group, indicating that AR induction altered the gut microbial community structure. Significant differences were also observed between the XQLD-treated group and the AR model group, as well as between the loratadine-treated group and the AR model group, suggesting that both interventions could partially modulate AR-associated alterations in gut microbiota structure. Based on the sample distribution in Figure 5C, the XQLD group exhibited a more distinct separation from the AR model group. However, the current statistical results are insufficient to conclude that the regulatory effect of XQLD on the gut microbiota is superior to that of loratadine.

Combined analysis of *α*-and *β*-diversity revealed distinct effects of XQLD on microbial communities at different sites along the gut–lung axis. In the nasal microbiota, the Faith’s phylogenetic diversity index showed a decreasing trend in the AR model group and was significantly increased following XQLD treatment. However, β-diversity analysis did not reveal a significant difference between the XQLD and AR groups, suggesting that the effect of XQLD on the nasal microbiota may be primarily reflected in the improvement of phylogenetic diversity, whereas its influence on the overall community structure appears limited. In the lung microbiota, neither α-diversity nor β-diversity indices showed significant differences among groups (*p* > 0.05), indicating that AR induction and XQLD intervention exerted minimal effects on the pulmonary microbial community. In contrast, significant differences in β-diversity were observed between the XQLD and AR groups in the gut microbiota (*p* < 0.05), whereas α-diversity indices remained relatively stable across groups. These findings suggest that XQLD exerts a more pronounced regulatory effect on the overall structure of the gut microbial community.

#### 3.2.4 Correlation analysis of nasal, lung, and gut microbiota with serum inflammatory factors

**Figure 6:**
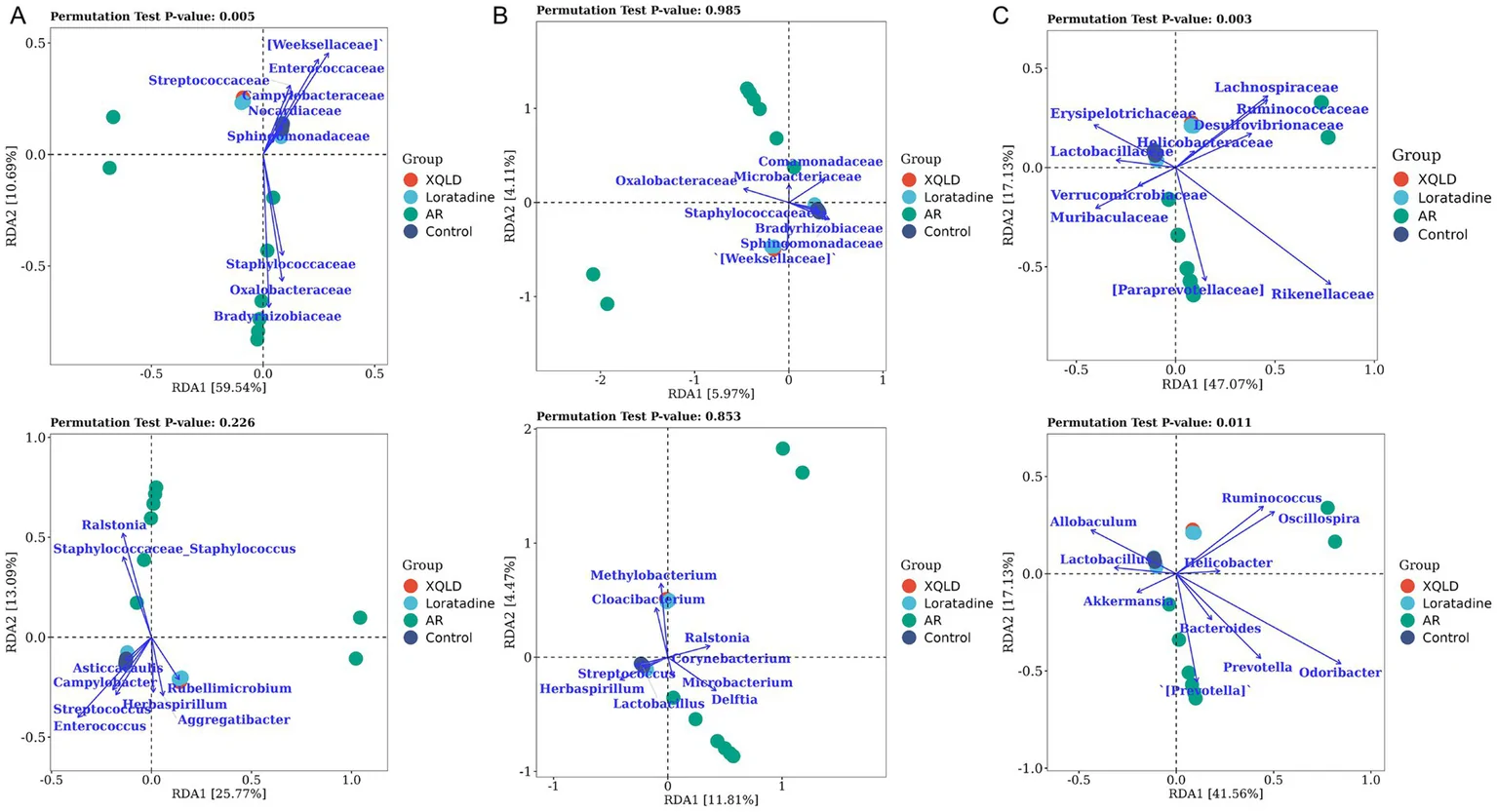
Redundancy analysis (RDA) of the mouse microbiota with inflammatory markers and nasal symptoms. **(A)** Nasal microbiota; **(B)** Lung microbiota; **(C)** Gut microbiota. Upper panel: Family level; lower panel: Genus level. Control: Control group; AR: AR model group; Loratadine: Loratadine group; XQLD: Xiaoqinglong Decoction group. The plot displays serum inflammatory factors (points) and microbial taxa (blue arrows). Arrow length indicates microbial impact magnitude; longer arrows suggest greater influence. Arrow angles reflect taxon-axis correlation, smaller angles denote higher correlation. Sample projections on arrows approximate corresponding factor values. *p*-values above show significance of microbial changes, smaller *p*-values indicate more impact. Percentages next to axes represent variance explained.

To explore the association between microbiota from different sites of the gut-lung axis and AR inflammatory factors as well as nasal symptoms, this study performed redundancy analysis (RDA) on the top 10 microbiota at the family and genus levels in relative abundance in the nasal, lung, and gut microbiota with serum cytokines and improvement of behavioral indicators (Figure 6). The results showed that the strength of association between the microbiota of the three sites and AR pathological changes differed significantly.

Among the nasal microbiota (Figure 6A), *Oxalobacteraceae* and *Ralstonia* were positively correlated with the control group and the treatment groups, but deviated in direction from the AR model group and inflammatory factors, suggesting that increased abundance of these microbiota is associated with alleviation of AR symptoms. The overall explanatory power of the lung microbiota was low (Figure 6B), and the associations of various microbiota with AR symptoms and inflammatory factors were weak, which was consistent with the results of the diversity analysis. The gut microbiota showed a stronger association (Figure 6C). *Muribaculaceae*, *Lactobacillaceae*, as well as *Lactobacillus* and *Allobaculum*, were highly positively correlated with the control group and the XQLD group, and were opposite in direction to inflammatory factors and nasal symptoms, indicating that the restoration of the abundances of the above-mentioned microbiota was closely associated with the alleviation of AR inflammation and improvement of behavioral indicators (*p* < 0.05). In summary, the strength of association between the microbiota of the three sites and AR pathological changes differed. The nasal microbiota showed a trend of partial correlation, the lung microbiota showed a weak association, whereas changes in the gut microbiota were most closely associated with the alleviation of AR inflammation and symptoms.

**Figure 7:**
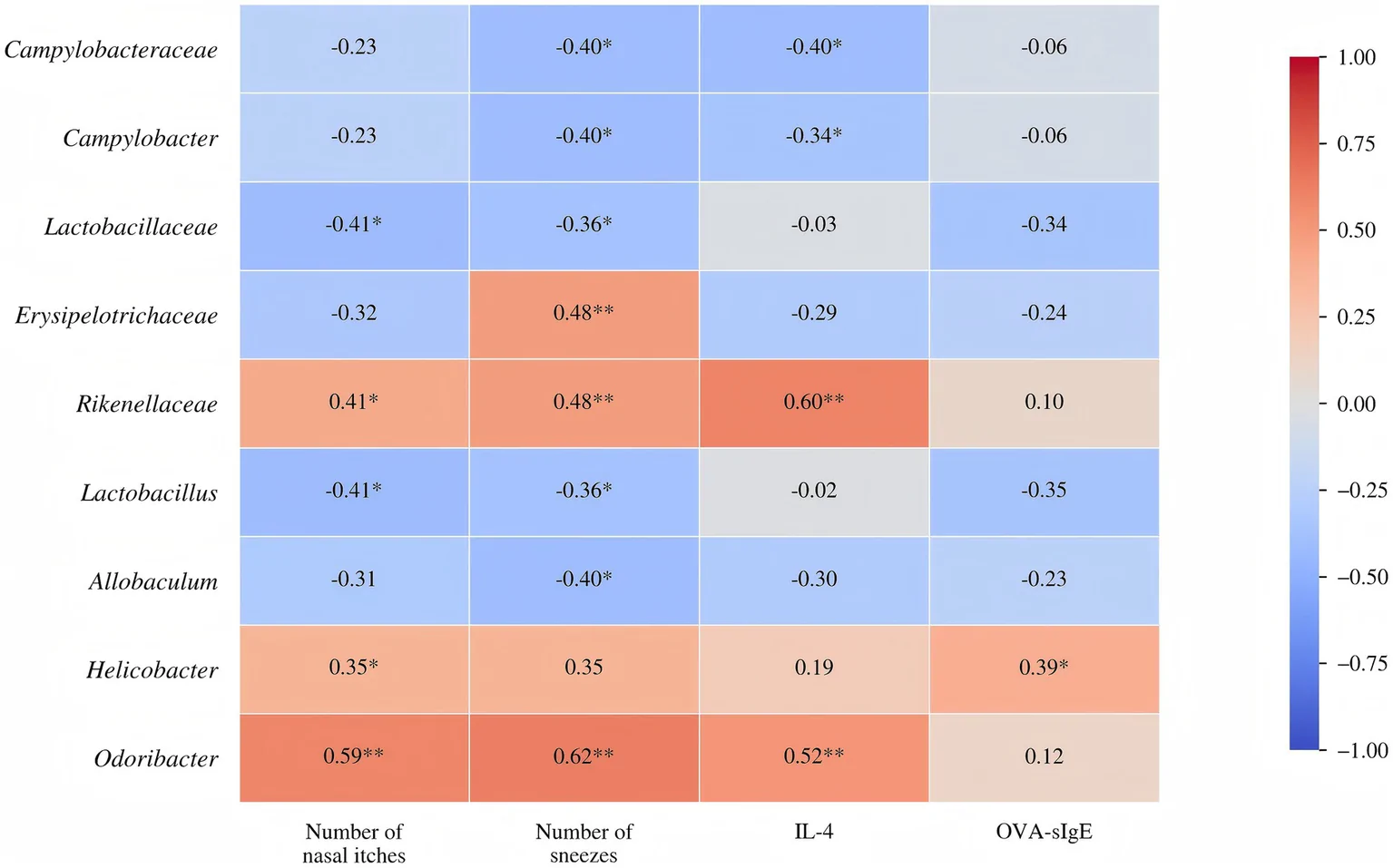
Spearman correlation heatmap showing the associations between the relative abundances of key nasal and gut microbial taxa and AR-related symptoms and inflammatory indicators. From left to right, the horizontal axis represents the number of nose-rubbing episodes, number of sneezes, serum IL-4 level, and serum OVA-sIgE level. The vertical axis represents the corresponding bacterial families or genera. Colors indicate the direction and magnitude of Spearman’s correlation coefficient, with red indicating positive correlations and blue indicating negative correlations; darker colors indicate stronger correlations. Values within the cells represent Spearman’s correlation coefficient (*r*), *w*ith *r* > 0 indicating a positive correlation and *r* < 0 indicating a negative correlation. Asterisks indicate statistical significance: ^*^p < 0.05 and ^**^p < 0.01.

To validate the associations between microbiota and AR inflammation revealed by the RDA analysis, this study performed Spearman correlation analysis on key microbiota in the nasal cavity and gut with nasal symptoms and inflammatory factors (Figure 7). The results showed that key microbiota in both the nasal cavity and gut were closely associated with AR severity, but the patterns of association showed some differences.

The relative abundance of nasal *Campylobacter* was significantly negatively correlated with the number of sneezes and serum IL-4 levels (*p* < 0.05). In the gut microbiota, *Lactobacillaceae* and *Lactobacillus* were significantly negatively correlated with the numbers of nose-rubbing episodes and sneezes, whereas *Allobaculum* was significantly negatively correlated with the number of sneezes (*p* < 0.05). *Erysipelotrichaceae* was significantly positively correlated with the number of sneezes (*p* < 0.01). *Rikenellaceae* was significantly positively correlated with the numbers of nose-rubbing episodes and sneezes and with serum IL-4 levels (*p* < 0.05). *Helicobacter* was significantly positively correlated with the number of nose-rubbing episodes and serum OVA-sIgE levels (*p* < 0.05), whereas *Odoribacter* was significantly positively correlated with the numbers of nose-rubbing episodes and sneezes and with serum IL-4 levels (*p* < 0.01). In summary, changes in the abundances of key genera in the nasal cavity and gut were closely associated with AR severity. Enrichment of beneficial bacteria and reduction of harmful bacteria were significantly correlated with alleviation of AR symptoms and downregulation of Th2-type inflammation. These results suggest that there is a clear association between the effects of XQLD on the gut-lung axis microbiota, particularly its promotion of the recovery of beneficial bacteria such as *Lactobacillaceae*/*Lactobacillus* and *Allobaculum* in the gut, and its therapeutic efficacy in alleviating AR symptoms.

#### 3.2.5 Prediction of functional changes in the nasal, lung, and gut microbiota

To explore the potential biological implications of the observed microbial community alterations, PICRUSt2, a functional prediction tool based on 16S rRNA gene sequencing, was used as a supplementary analysis following the diversity and correlation analyses (Langille et al., 2013). The analysis focused on predicted functional pathways associated with differential microbial taxa that were statistically correlated with nasal symptoms or immunological indicators. These predictions were used to generate hypotheses for subsequent mechanistic studies rather than to demonstrate actual metabolic activity.

The analysis indicated that, after AR modeling, the predicted functions of the microbiota in different sites changed. In the nasal microbiota, *Campylobacter*, which was negatively correlated with symptoms, is generally associated with Th1-type immune activation. Its decreased abundance suggests that the local immune environment of the nasal cavity may shift from a mixed Th1/Th17 response to a pure Th2 polarization, which is consistent with the typical pathological features of AR. In the gut microbiota, the functional changes were more systematic and their association with symptoms was more explicit. On the one hand, the predicted functions of beneficial bacteria positively correlated with healthy status were primarily immunomodulation and barrier maintenance. *Lactobacillus* and *Allobaculum*, whose abundances are negatively correlated with symptoms, are both key producers of SCFAs. SCFAs are known immunomodulatory metabolites, and the literature reports that they have potential functions including activating G protein-coupled receptors, inhibiting histone deacetylases, promoting regulatory T cell (Treg) differentiation, suppressing Th2 responses, and enhancing epithelial barrier integrity (Smith et al., 2013). Similarly, *Rikenellaceae* and *Odoribacter* are also involved in the production of SCFAs or aryl hydrocarbon receptor (AhR) ligands, metabolites that play important roles in maintaining immune tolerance (Hiippala et al., 2018; Parker et al., 2020). These beneficial metabolic functions showed a decreasing trend during AR modeling. On the other hand, bacteria positively correlated with symptoms are associated with pro-inflammatory or metabolic dysfunction functions. Meanwhile, the enrichment of *Erysipelotrichaceae* is often associated with metabolic disorders (Kaakoush, 2015). These changes suggest that during AR modeling, the functional balance of the gut microbiota may be disrupted, manifesting as an enhancement of pro-inflammatory and damage-related functions, along with a weakening of protective and anti-inflammatory metabolic functions.

In the XQLD group, predicted pathways related to SCFA metabolism changed in a direction consistent with partial reversal of the AR-associated profile, whereas some inflammation-related pathways showed downward trends. The loratadine group also exhibited changes in predicted functional profiles, although the overall descriptive trend appeared less pronounced than that in the XQLD group. Because these findings were inferred from 16S rRNA gene sequencing rather than directly measured metabolites or functional activity, comparisons between the two interventions should be interpreted cautiously.

## 4 Discussion

Based on our previous finding that XQLD has a regulatory effect on the gut microbiota in AR mice (Liu et al., 2024), the present study further investigated whether XQLD also affects the microbiota of other key sites of the gut-lung axis, whether there are differences in its effects, and whether its effect on the gut-lung axis microbiota correlates with its efficacy in alleviating AR symptoms. The results showed that XQLD effectively alleviated nasal allergic symptoms and inflammatory responses and repaired nasal mucosal tissue damage in AR mice (Figure 1). The study found that the therapeutic effect of XQLD on AR is associated with its influence on the microbiota of different sites of the gut-lung axis, and that the response patterns of microbiota in different sites to the AR modeling and drug intervention are significantly different.

The lung microbiota did not show significant changes in this study. Alpha and beta diversity analyses showed that neither the AR model group nor the drug intervention induced significant changes in the structure of the lung microbiota (Figures 4,5). At the family and genus levels, *Oxalobacteraceae* and *Ralstonia* were the dominant taxa in the lung microbiota, and their relative abundances remained stable among groups (Figure 3). Redundancy analysis also suggested that the lung microbiota was weakly associated with AR symptoms and inflammatory parameters (Figure 6). The healthy lung is a low-biomass environment, where the microbial community is under stringent regulation by mucociliary clearance, the mucosal barrier, and host immune clearance mechanisms (Kaakoush, 2015). Moreover, as AR primarily involves the upper respiratory tract, the absence of significant alterations in the lung microbiota may reflect the ability of the host immune system to limit the spread of upper airway inflammation. These findings contrast with the pronounced changes observed in the nasal and gut microbiota, further highlighting the heterogeneity of distinct microecological niches along the gut–lung axis during AR pathogenesis and XQLD intervention.

Unlike the lung, the nasal microbiota exhibited significant structural changes after AR modeling. This was mainly manifested as a decrease in the abundance of *Oxalobacteraceae*, a core beneficial family, from 65% in the control group to 35% in the AR model group, along with decreasing trends in the Shannon index and Faith index (Figure 4). Among them, the overall species richness of the nasal microbiota was only 489 OTUs, which was much lower than the 14,234 OTUs of the gut microbiota (Figure 2), indicating that as an open niche, the nasal cavity has a relatively simple microbial composition, making it more susceptible to direct impact from the local inflammatory environment, mucus secretion, and foreign antigens. Because nasal and lung samples are characterized by low microbial biomass, contamination screening was performed using the decontam frequency method. The major community metrics and taxonomic profiles at the family and genus levels remained essentially unchanged after filtering, suggesting that potential contamination had a limited effect on the principal findings. Correlation analysis showed that the relative abundance of nasal *Campylobacter* was significantly negatively correlated with the number of sneezes and serum IL-4 levels (Figure 7). Certain species of the genus *Campylobacter* can activate Th1/Th17-type immune responses under specific conditions. The decrease in their abundance suggests that under AR conditions, the local immune environment of the nasal cavity may be shifting from a mixed response toward Th2 polarization, a change that is consistent with the typical pathological features of AR. The changes in the nasal microbiota may be induced by local inflammation and, in turn, may affect the immune status of the nasal cavity. This interaction pattern differs from the mode of action by which the gut microbiota systemically influences host immune responses.

In contrast, the changes in the gut microbiota are more directly associated with the pathological state of AR. Beta diversity analysis showed that there was a significant difference in the gut microbiota between the AR model group and the control group, indicating that AR modeling induced changes in the gut microbiota (Figure 5). In terms of intervention effects, the effect of XQLD on the gut microbiota was more pronounced than that of loratadine. The number of unique OTUs in the gut of the XQLD group reached 3,659, which was higher than the 2,833 in the loratadine group (Figure 2). Beta diversity analysis also showed that the sample points of the XQLD group were clearly separated from those of the AR model group, whereas there was still considerable overlap between the loratadine group and the AR model group (Figure 5). These results hint that XQLD may exert a relatively greater influence on the overall structure of the gut microbiota compared with loratadine under the present experimental conditions.

Further correlation analysis and functional prediction analysis indicated that changes in the gut microbiota are directly associated with the immunopathological mechanism of AR. The relative abundances of health-associated bacterial taxa, including *Lactobacillaceae*, *Lactobacillus*, and *Allobaculum*, were negatively associated with nasal symptoms and inflammatory cytokine levels. (Figure 7). These bacteria are generally considered to be involved in the production of SCFAs (Smith et al., 2013; Li et al., 2018), which contribute to immune regulation and intestinal barrier maintenance. Although *Rikenellaceae* has been reported to participate in SCFA-related metabolic processes and immune regulation, its correlation pattern in the present study differed from other beneficial-associated taxa, showing a positive correlation trend with certain inflammatory indicators. Therefore, the specific role of *Rikenellaceae* in AR progression requires further investigation. In contrast, the abundances of *Erysipelotrichaceae* and *Helicobacter* were significantly positively correlated with nasal allergic symptoms and serum inflammatory factor levels in mice. Enrichment of *Erysipelotrichaceae* is often associated with metabolic dysregulation and a pro-inflammatory state, and can exacerbate immune responses by activating inflammatory pathways; certain species of the genus *Helicobacter* can disrupt the epithelial barrier, promote the release of inflammatory factors, and trigger local inflammatory reactions. Similar to the aforementioned beneficial bacteria, changes in the abundance of these harmful bacteria are closely associated with the pathological state of AR, and their increase may weaken the protective immune function of the gut and nasal cavity, thereby exacerbating AR symptoms. In the AR model group, the abundance of *Lactobacillaceae* decreased from 11.6 to 8.5%, and *Lactobacillus* and *Allobaculum* showed a similar decreasing trend; whereas the abundances of symptom-positively correlated bacteria such as *Erysipelotrichaceae* and *Helicobacter* increased from 2.3 to 5.8%. This trend of decreasing beneficial bacteria and increasing harmful bacteria suggests that after AR modeling, the functional balance of the gut microbiota is disrupted, manifesting as a weakening of protective and anti-inflammatory metabolic functions, accompanied by an enhancement of pro-inflammatory and damage-related functions.

In the XQLD group, the abundance of *Lactobacillaceae* in the gut microbiota rebounded to 11.6%, and *Lactobacillus* and *Allobaculum* were also restored (Figure 3). The decrease in the abundance of these beneficial bacteria is likely associated with the weakening of anti-inflammatory metabolic function in AR. After XQLD intervention, the above-mentioned functional imbalance of the microbiota showed a clear trend of reversal, with the abundance of SCFA-producing microbiota increasing. Meanwhile, in the gut of the XQLD group, the abundance of *Erysipelotrichaceae* decreased to 2.1%, and the decrease in these potential pathogenic bacteria occurred simultaneously with the improvement of nasal allergic symptoms and inflammatory markers in mice. In contrast, in the loratadine group, the abundances of *Erysipelotrichaceae* and *Helicobacter* decreased only slightly to 4.8 and 5.2%, respectively, and the magnitude and specificity of gut microbiota regulation were weaker than those in the XQLD group. As a selective H1 receptor antagonist, loratadine primarily acts on the terminal step of histamine release, and its effect on the gut microbiota is relatively indirect and limited. As a compound Chinese herbal medicine, the multi-component, multi-target characteristics of XQLD may confer a broader regulatory effect on the microbiota, which helps to explain its more sustained therapeutic efficacy and lower tendency for relapse in clinical application.

Considering the changes in microbiota across multiple sites of the gut-lung axis, although there were significant differences between the nasal and gut microbiota in terms of diversity levels, absolute abundance, and magnitude of change, this study found that the two showed consistency in the changing trends of key beneficial bacteria. In the AR model group, the relative abundance of *Oxalobacteraceae* in the nasal cavity decreased from 65 to 35%, while that of *Lactobacillaceae* in the gut decreased from 11.6 to 8.5%, indicating a reduction in both taxa. Following XQLD treatment, their relative abundances increased to 40 and 11.6%, respectively (Figure 3). Such cross-site synergistic changes are difficult to explain solely by local microenvironmental factors but rather point more likely to a systemic regulatory network, namely the gut-lung axis. Previous studies have suggested that metabolites or immune cells derived from the gut microbiota may migrate via the circulatory system to distant mucosal sites and potentially influence the immune status of the respiratory tract and nasal cavity (Wang et al., 2014; Zhang et al., 2020). In this study, after XQLD intervention, the changes in the gut and nasal microbiota showed the above-mentioned synchrony, and the degree of recovery of the nasal microbiota was smaller than that of the gut microbiota. This may be related to the fact that the nasal cavity, as an open niche, is more susceptible to interference from external environmental factors. It may also suggest that the direct effect of XQLD on the gut microbiota is more pronounced, and that the direct regulatory effect of XQLD on the gut mthe localiota may indirectly affect the nasal microbiota and local immune environment through the systemic pathway of the gut-lung axis, thereby exerting a synergistic anti-AR effect, although further investigation is warranted to delineate the specific signaling pathways involved.

## 5 Conclusion

This study systematically investigated the effects of XQLD on the microbiota of three key sites of the gut-lung axis, namely the nasal cavity, lung, and gut, in AR mice, as well as the differential responses of the microbiota at each site, and analyzed the association between this differential regulation and recovery from AR. The study showed that the response patterns of the microbiota in the three sites to AR modeling and XQLD intervention were distinctly different. The lung microbiota showed no significant change and remained stable. The nasal microbiota primarily exhibited a decrease in the abundance of core beneficial bacteria and a decrease in diversity, with local structural changes. The gut microbiota showed the most pronounced changes, primarily structural remodeling, with significantly altered beta diversity, and the separation between the XQLD group and the AR model group was the most obvious. This study also found that the nasal and gut microbiota showed a certain cross-site synchrony in changes of key beneficial bacteria, suggesting the existence of a systemic regulatory mechanism of the gut-lung axis. The regulatory effect of XQLD on the gut microbiota appeared to be more pronounced than that of loratadine under the present experimental conditions. This difference might be related to the multi-component, multi-target characteristics of the compound formula, a feature that may also help to explain the more sustained therapeutic efficacy and lower relapse tendency observed with XQLD in clinical practice. Given that this study is based on 16S rRNA sequencing data, further studies are needed to directly verify changes in gut microbiota metabolites using metagenomic sequencing or metabolomics techniques, and to establish the causal relationship between microbiota and therapeutic efficacy through fecal microbiota transplantation experiments, in order to further elucidate the specific mechanism by which XQLD treats AR via the gut-lung axis.

## Data Availability

The data presented in the study are deposited in the NCBI repository, accession number PRJNA1472906.

## Footnotes

1
https://powo.science.kew.org/

2
https://www.genescloud.cn
