# Supplementary material for: Effect of Xiaoqinglong decoction on gut–lung axis microbiota in allergic rhinitis mice
Source: Front Microbiol. 2026 Aug 19;17:1917709. doi: 10.3389/fmicb.2026.1917709 (PMC13534138; doi:10.3389/fmicb.2026.1917709)
Supplement: Supplementary file 1 [file Supplementary_file_1.docx]

**Supplementary Material 1-1:** Flowchart of mouse model establishment and drug administration.

On days 0, 7, and 14, mice in all groups except the blank control group were intraperitoneally injected with 200 μL of normal saline containing 50 μg OVA (Sigma-Aldrich, USA; Cat. No.: A5503) and 4 mg aluminum hydroxide (Sigma-Aldrich, USA; Cat. No.: V900163) for systemic sensitization, while the blank control group received an equal volume of normal saline. From days 22 to 28, intranasal challenge was performed once daily in all groups except the blank control group. Each nostril was administered 10 μL of OVA solution (100 μg/μL, dissolved in normal saline), whereas the blank control group received an equal volume of normal saline. On day 29, mice were euthanized by cervical dislocation following overdose anesthesia, and samples were collected.

Meanwhile, from days 15 to 28, mice in each treatment group were administered the corresponding drug by gavage 1 h prior to intranasal challenge. The XQLD group received 100 μL of XQLD solution (2.1 g/mL), while the loratadine group received 100 μL of loratadine solution (0.278 mg/mL; loratadine tablets, Bayer Healthcare (Shanghai) Co., Ltd., China; Approval No.: H10970410). The blank control and AR model groups received an equal volume of normal saline.

Days 0-14 involve three basic sensitizations through intraperitoneal injection. Days 15-28 include daily drug intervention therapy on the stomach. Days 22-28 involve intranasal administration for nasal sensitization. Day 29 is the time for sampling. i.p.: intraperitoneal injection; OVA: ovalbumin; Alum: aluminum hydroxide; Intranasal ad.: intranasal administration; i.g.: intragastric administration.

**Supplementary Material 1-2:** Chinese medicinal herbs contained in Xiaoqinglong decoction and the supplier information of all herbs.

| **Chinese naming of different ingredients** | **Full botanical plant names** | **Amount (g)** | **Manufacturer source** | **Lot Number** |
| --- | --- | --- | --- | --- |
| Zhi Gan Cao | *Glycyrrhiza uralensis* Fisch*.* | 9 | Kangmei Pharmaceutical Co., Ltd. | 220902211 |
| Ma Huang | *Ephedra sinica* Stapf | 9 | Kangmei Pharmaceutical Co., Ltd. | 220801 |
| Bai Shao | *Paeonia lactiflora* Pall*.* | 9 | Kangmei Pharmaceutical Co., Ltd. | 220900199 |
| Wu Wei Zi | *Schisandra Chinensis* (Turcz.) Baill. | 12 | Kangmei Pharmaceutical Co., Ltd. | 220802061 |
| Gan Jiang | *Zingiber officinale* Roscoe | 9 | Kangmei Pharmaceutical Co., Ltd. | 220701381 |
| Gui Zhi | *Neolitsea cassia* (L.) Kosterm | 9 | Kangmei Pharmaceutical Co., Ltd. | 220801781 |
| Xi Xin | *Asarum heterotropoides F. Schmidt* | 9 | Kangmei Pharmaceutical Co., Ltd. | 220701471 |
| Fa Ban Xia | *Pinellia ternata* (Thunb.) Makino | 12 | Kangmei Pharmaceutical Co., Ltd. | 22030202 |

**Supplementary Material 1-3A:** Candidate contaminant ASVs identified using the decontam frequency method at *p*_freq < 0.10.

| Site | ASV ID | *p_freq_* | Detected samples, n | Reads, n (%) | Taxonomic annotation (family; genus; species) |
| --- | --- | --- | --- | --- | --- |
| Nasal | ASV_636 | 0.078373 | 3 | 11 (0.000487) | *Lactobacillaceae*; *Lactobacillus*; *Lactobacillus helveticus* |
| Nasal | ASV_225 | 0.016268 | 2 | 6 (0.000266) | unidentified *Burkholderiales* (family, genus, species) |
| Lung | ASV_887 | 0.012505 | 2 | 5 (0.000141) | *Clostridiaceae*; *Clostridium*; *Clostridium perfringens* |
| Lung | ASV_554 | 0.037858 | 3 | 5 (0.000141) | *Lactobacillaceae*; *Lactobacillus*; unidentified *Lactobacillus* |
| Lung | ASV_509 | 0.091071 | 2 | 12 (0.000339) | *Rhizobiaceae*; unclassified *Rhizobiaceae* (genus and species) |
| Lung | ASV_247 | 0.063886 | 2 | 8 (0.000226) | *Streptococcaceae*; *Streptococcus*; unidentified *Streptococcus* |

**Supplementary Material 1-3B:** Summary of decontam filtering and sensitivity analyses for nasal and lung samples.

| Site | Input dataset | Candidate ASVs and reads by *p_freq_* threshold | Sequence retention at *p_freq_* < 0.10 | Changes in community metrics at *p_freq_* < 0.10 |
| --- | --- | --- | --- | --- |
| Nasal | 32 samples; 538 ASVs; 2,259,416 reads; DNA 129.5-525.8 ng/μL | 0.10: 2 ASVs (0.372%); 17 reads (0.000752%) 0.20: 7 ASVs (1.301%); 373 reads (0.016509%) 0.50: 31 ASVs (5.762%); 1,601,660 reads (70.888230%) | Mean 99.999290%; minimum 99.991558% | Observed ASVs: max Δ 1; Shannon: max \|Δ\| 0.000827; Bray-Curtis: max \|Δ\| 0.000062; family/genus mean: max Δ 0.001523 percentage points |
| Lung | 32 samples; 968 ASVs; 3,541,313 reads; DNA 442.5-1593.2 ng/μL | 0.10: 4 ASVs (0.413%); 30 reads (0.000847%) 0.20: 8 ASVs (0.826%); 257 reads (0.007257%) 0.50: 71 ASVs (7.335%); 54,336 reads (1.534346%) | Mean 99.999201%; minimum 99.991450% | Observed ASVs: max Δ 2; Shannon: max \|Δ\| 0.000911; Bray-Curtis: max \|Δ\| 0.000082; family/genus mean: max Δ 0.002462 percentage points |

**Supplementary Material 1-4: Relative abundances and statistical comparisons of selected bacterial taxa.**

| **Site** | **Taxonomic** **level** | **Bacterial taxon** | **Relative abundance** **in AR group (%)** | **Relative abundance** **in XQLD group (%)** | ***p* value** | ***q* value** |
| --- | --- | --- | --- | --- | --- | --- |
| Nasal | Family | *Oxalobacteraceae* | 34.97 | 39.95 | 0.0009 | 0.0094 |
| Lung | Family | *Oxalobacteraceae* | 85.25 | 96.06 | 0.6365 | 0.8752 |
| Gut | Family | *Lactobacillaceae* | 8.50 | 11.61 | 0.0135 | 0.0676 |
| Gut | Family | *Rikenellaceae* | 3.85 | 1.62 | 0.001359 | 0.013594 |
| Nasal | Genus | *Ralstonia* | 47.13 | 43.21 | 0.9581 | 1.0000 |
| Nasal | Genus | *Campylobacter* | 0.98 | 6.75 | 0.0486 | 0.4860 |
| Lung | Genus | *Ralstonia* | 85.08 | 95.02 | 0.4948 | 0.8425 |
| Lung | Genus | *Microbacterium* | 12.41 | 0.00 | 0.0128 | 0.1278 |
| Gut | Genus | *Lactobacillus* | 8.49 | 8.94 | 0.7929 | 0.9581 |
| Gut | Genus | *Allobaculum* | 1.41 | 2.76 | 0.4309 | 0.9581 |
| Gut | Genus | *Helicobacter* | 2.39 | 2.21 | 0.3720 | 0.9581 |
| Gut | Genus | *Odoribacter* | 2.21 | 0.88 | 0.001948 | 0.019475 |

**Supplementary Material 1-5:** List of English Abbreviations

| **English Abbreviation** | **English Full Term** |
| --- | --- |
| AR | Allergic Rhinitis |
| XQLD | Xiaoqinglong Decoction |
| OVA | Ovalbumin |
| IgE | Immunoglobulin E |
| IL-4 | Interleukin-4 |
| SCFAs | Short-Chain Fatty Acids |
| OTU | Operational Taxonomic Unit |
| ELISA | Enzyme-Linked Immunosorbent Assay |
| 16S rRNA | 16S Ribosomal RNA |
| LC-MS | Liquid Chromatography-Mass Spectrometry |
| RDA | Redundancy Analysis |
| ANOSIM | Analysis of Similarities |
| PCR | Polymerase Chain Reaction |
| GPR43 | G Protein-Coupled Receptor 43 |
| GPR109A | G Protein-Coupled Receptor 109A |
| HDAC | Histone Deacetylase |
| AhR | Aryl Hydrocarbon Receptor |
| Treg | Regulatory T cell |
| Th1 | T helper 1 cell |
| Th2 | T helper 2 cell |
| Th17 | T helper 17 cell |
| KEGG | Kyoto Encyclopedia of Genes and Genomes |
